# Environmental Controls on Multi-Scale Dynamics of Net Carbon Dioxide Exchange From an Alpine Peatland on the Eastern Qinghai-Tibet Plateau

**DOI:** 10.3389/fpls.2021.791343

**Published:** 2022-01-05

**Authors:** Hu Yao, Haijun Peng, Bing Hong, Qian Guo, Hanwei Ding, Yetang Hong, Yongxuan Zhu, Cheng Cai, Jinshu Chi

**Affiliations:** ^1^State Key Laboratory of Environmental Geochemistry, Institute of Geochemistry, Chinese Academy of Sciences, Guiyang, China; ^2^Bayinbuluk Alpine Wetland Carbon Flux Research Station, Chinese Flux Observation and Research Network, Beijing, China; ^3^University of Chinese Academy of Sciences, Beijing, China; ^4^CAS Center for Excellence in Quaternary Science and Global Change, Xi’an, China; ^5^School of Chemical Engineering, Guizhou Institute of Technology, Guiyang, China; ^6^Department of Forest Ecology and Management, Swedish University of Agricultural Sciences, Umeå, Sweden

**Keywords:** NEE, eddy covariance, alpine peatland, wavelet analysis, Qinghai-Tibetan Plateau

## Abstract

Peatlands are characterized by their large carbon storage capacity and play an essential role in the global carbon cycle. However, the future of the carbon stored in peatland ecosystems under a changing climate remains unclear. In this study, based on the eddy covariance technique, we investigated the net ecosystem CO_2_ exchange (NEE) and its controlling factors of the Hongyuan peatland, which is a part of the Ruoergai peatland on the eastern Qinghai-Tibet Plateau (QTP). Our results show that the Hongyuan alpine peatland was a CO_2_ sink with an annual NEE of −226.61 and −185.35 g C m^–2^ in 2014 and 2015, respectively. While, the non-growing season NEE was 53.35 and 75.08 g C m^–2^ in 2014 and 2015, suggesting that non-growing seasons carbon emissions should not be neglected. Clear diurnal variation in NEE was observed during the observation period, with the maximum CO_2_ uptake appearing at 12:30 (Beijing time, UTC+8). The Q_10_ value of the non-growing season in 2014 and 2015 was significantly higher than that in the growing season, which suggested that the CO_2_ flux in the non-growing season was more sensitive to warming than that in the growing season. We investigated the multi-scale temporal variations in NEE during the growing season using wavelet analysis. On daily timescales, photosynthetically active radiation was the primary driver of NEE. Seasonal variation in NEE was mainly driven by soil temperature. The amount of precipitation was more responsible for annual variation of NEE. The increasing number of precipitation event was associated with increasing annual carbon uptake. This study highlights the need for continuous eddy covariance measurements and time series analysis approaches to deepen our understanding of the temporal variability in NEE and multi-scale correlation between NEE and environmental factors.

## Introduction

Peatlands worldwide have been shown to be an important player in the global carbon cycle in the recent past (Page and Baird, 2016; Helbig et al., 2019; D’Angelo et al., 2021). Even though they cover only approximately 3% of the global land area, the carbon stored in their soils has been estimated to be more than 600 Pg (1 Pg = 10^15^ g) worldwide since the Last Glacial Maximum (Yu et al., 2010). The carbon storage in peatlands accounts for about one third of the total global soil carbon pool (Wilson et al., 2016). It is generally thought that the intensity of carbon sinks in peatlands is higher than that in other terrestrial ecosystems due to primary production exceeding decomposition and other losses (León and Oliván, 2014). However, the carbon sink function of peatlands can be substantially altered due to climate warming, land-use change, and other human disturbances (Ward et al., 2012; Lupascu et al., 2014). Thus, a profound understanding on spatiotemporal variation characteristics of the carbon flux in peatlands and how the flux responds to its controlling factors is vital for the global carbon cycle research.

The Qinghai-Tibet Plateau (QTP), known as “the third pole”, occupies approximately 2.5 × 10^6^ km^2^ with an average elevation of over 4,000 m (Zhang et al., 2014). The eastern part of QTP is more vulnerable to climate change due to the superimposed influence of the Indian summer monsoon, Eastern Asian summer monsoon, and westerly circulation (Kang et al., 2010). In recent years, several studies into the alpine wetland CO_2_ exchange and its environmental factors have been conducted on the QTP (Peng et al., 2015; Wang et al., 2016; Kang et al., 2018). However, the magnitude of the carbon budgets, exchange, and direction (net sink/absorption or net source/emission) varies with temporal dynamics. Some studies suggested that the alpine wetland on the QTP was a huge organic carbon sink that was highly sensitive to global climate change (Hao et al., 2011; Cao et al., 2017; Niu et al., 2017). But other studies identified the alpine wetland on the QTP as a carbon source (Zhang et al., 2008; Zhao et al., 2010; Zhu et al., 2020). In addition, the main environmental factors that affect the carbon source/sink functions of the alpine wetland on the QTP have not been clearly understood. Many studies (e.g., Aslan-Sungur et al., 2016; Kang et al., 2018; D’Angelo et al., 2021) showed that wetland CO_2_ fluxes are affected by a variety of ecological factors, such as temperature, soil water content, and solar radiation. Furthermore, some studies (Ali et al., 2008; Wu et al., 2020) suggested that whether alpine wetland acted as a CO_2_ sink or a CO_2_ source depended on the length of the growing season. Overall, there is no consensus as to the carbon source or sink of wetland ecosystems on the QTP due to different environmental factors in different wetland ecosystems. Besides, previous studies mainly focused on the growing season, making it difficult to fully explain the dynamics of wetland ecosystem carbon exchange and its impact mechanism. Therefore, a long-term and continuous field observation is needed to better understand the dynamic changes of carbon exchange of alpine wetland ecosystems (Griffis et al., 2000; Song et al., 2011).

Most studies on carbon exchange on the QTP were carried out by the traditional static chamber method (Wang et al., 2007; Pei et al., 2009; Wei et al., 2012). But Koskinen et al. (2014) pointed out that the installation of gas chambers may change the micrometeorological environment of the measured area and the gas diffusion gradient in the soil profile. The eddy covariance (EC) method provides reliable flux measurements (Zhao et al., 2010) and has been widely used to measure CO_2_ fluxes in terrestrial ecosystem (Aubinet et al., 2000; Yamamoto et al., 2001). It could not only offer a direct signal of CO_2_ fluxes but also record continuous NEE, which is ideally suited for time series analysis. Wavelet analysis has been demonstrated to be a powerful data analysis method to reveal the temporal variability in NEE and its dependencies on environmental control factors (e.g., Stoy et al., 2005; Furon et al., 2008; Jia et al., 2018). In this study, the EC method was used to measure the NEE between an alpine peatland ecosystem and the atmosphere in 2014 and 2015. Based on the quasi-continuous half-hour CO_2_ fluxes, the temporal dynamics of alpine peatland CO_2_ exchange and its environmental controls on time-scales ranging from hourly to annually were investigated.

The main objectives of this paper are to (1) investigate the CO_2_ sequestration potential of alpine peatland ecosystem on the QTP; (2) assess the diurnal and seasonal variations in NEE, and (3) explore the effects of environmental factors on NEE at different timescales. Based on preliminary research, we hypothesize that (1) Hongyuan peatland is a CO_2_ sink and has a greater potential for CO_2_ sequestration capacity compared with other alpine meadow and alpine steppe ecosystems at similar elevation and latitude on the QTP, (2) NEE exhibited clear diurnal and seasonal variations, and (3) photosynthetically active radiation, soil temperature, and precipitation are the main environmental factors that affect NEE variations on the daily, seasonal, and interannual timescales, respectively.

## Materials and Methods

### Site Description

**FIGURE 1:**
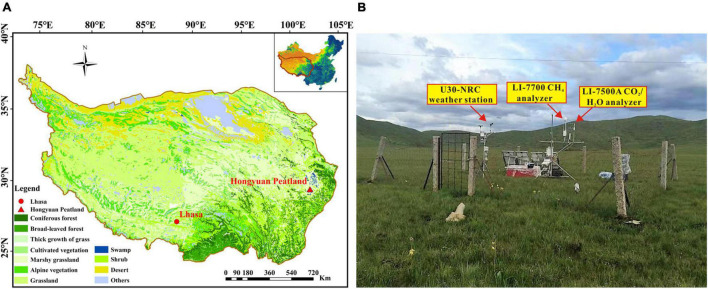
**(A)** Vegetation types on the QTP. The red triangle represents the location of the site. The data was retrieved from the Data Center for Resources and Environmental Sciences of Chinese Academy of Sciences (http://www.csdb.cn); **(B)** Landscape and instruments at the Hongyuan Peatland Carbon Flux Monitoring and Research Station.

The study was carried out at the Hongyuan Peatland Carbon Flux Monitoring and Research Station (32°46′ N, 102°30′ E; 3,510 m above sea level), which was established by the Institute of Geochemistry, Chinese Academy of Sciences. The Hongyuan peatland is a part of the Ruoergai peatland located on the eastern QTP (Figure 1A), and is characterized by wet and humid summers with cool and dry winters. The mean annual precipitation and air temperature in this area is 746 mm and 1.8°C, respectively, according to the meteorological data (1981 ∼ 2010) from the Nation Benchmark Climate Station in Hongyuan^1^. More than 75% of annual precipitation occurs during May and September. The dominant vegetation species are *Carex mulieensis* and *Kobresia tibetica* with an average height of about 40 cm during the growing season. Before the installation of the research station, the spatial distribution of the peatland was investigated using a MALÅ ProEx ground penetrating radar (MALÅ Geosciences, Sweden) and a Russian Peat Corer. The results showed that the depth of Hongyuan peatland ranges from 0.5 and 6.5 m. The total area of the Hongyuan peatland was measured to be 1.1 km^2^. The EC tower was installed in the center of the Hongyuan peatland, where the terrain is flat and serves as an ideal place for micrometeorological flux measurements. The flux footprint analysis indicated that 90% of the CO_2_ flux originated from an area within 200 m of the EC tower, demonstrating that the measured fluxes are representative of the Hongyuan peatland area.

### Flux Measurements

The eddy covariance system consists of a Gill WindMaster (Pro) three-dimensional (3-D) ultrasonic anemometer (Gill Instruments Ltd., Lymington, Hampshire, United Kingdom), which measures 3-D wind velocities and virtual air temperatures, and an open path infrared CO_2_/H_2_O gas analyzer (LI-7500A, LI-COR, United States), which measures concentrations of CO_2_ and water vapor. The EC system was installed on a tripod tower 2.5 m above canopy (Figure 1B). The height of the EC system above ground was determined by the maximum height of vegetation during the growing season. To avoid water accumulation on the lens, the LI-7500A sensor was tilted 10° in the main wind direction. The raw data were recorded at a frequency of 10 Hz and stored as separate files by a data logger (LI-COR Inc., United States).

### Environmental Factors

Meteorological variables, including air temperature (Ta), soil temperature (Ts), soil water content (SWC), global radiation (Rg), relative humidity (RH) and precipitation (PPT), were monitored on every half-hour basis using a HOBO U30-NRC (Onset Computer Corporation, United States) weather station installed near the EC tower (Figure 1). Ts was measured at depths of 10 cm, 25 cm, and 40 cm below the ground. Ta was measured at a height of 2 m above the ground, with a 12-Bit Temp Smart Sensor (S-TMB-M006, Onset Computer Corporation, United States). Vapor pressure deficit (VPD) was calculated using the Ta and RH measurements. SWC was measured at a depth of 10 cm below the ground using a 10 HS Soil Moisture Smart Sensor (S-SMD-M005, Onset Computer Corporation, United States). Wind direction and speed were measured using smart sensors (S-WSA-M003, Onset Computer Corporation, United States; S-WDA-M003, Onset Computer Corporation, United States). Solar radiation was measured using a silicon pyranometer sensor (S-LIB-M003, Onset Computer Corporation, United States). PPT was measured using a rainfall smart sensor (S-RGB-M002, Onset Computer Corporation, United States). Water table level was observed using a ZKGD3000-M digital water level gauge (Beijing Zhongke Guangda Automation Technology, China) in a 2-m deep hydrological well near the EC tower.

### Data Processing

The EddyPro software version 6.2.0 (LI-COR, Inc., United States) was used to process raw data to obtain 30-min fluxes for analysis and recording. The main calculation procedures include cross wind correction of the sonic temperature (Liu et al., 2001), double coordinate rotation (Wilczak et al., 2001), Webb-Pearman-Leuning (WPL) correction (Webb et al., 1980), time lag compensation, statistical testing (Vickers and Mahrt, 1997), spectral correction (Moncrieff et al., 1997) and a footprint analysis (Kljun et al., 2004).

After the data-processing in EddyPro, the following steps were used to filter out the abnormal flux data based on sensor malfunctions and stable atmospheric conditions. First, poor quality fluxes with flag 2 (bad quality) were discarded (Foken et al., 2004). Second, in order to avoid the underestimation of nighttime NEE under calm climate conditions, the friction velocity (u*) threshold was determined by the BGC-jean online gap-filling tool (BGC16, Sect. 3) (Wutzler et al., 2018). In this study, all CO_2_ fluxes were excluded from analysis when u* < 0.091 m s^–1^. Finally, an outlier detection method was used to filter out occasional spikes based on the method reported in Papale et al. (2006). In this method, each 30 min CO_2_ flux *NEE*_*i*_ corresponds to a value *d*_*i*_, which is calculated as:


(1) $$d_{i}=\left(N\,E\,E_{i}-N\,E\,E_{i-1}\right)-\left(N\,E\,E_{i+1}-N\,E\,E_{i}\right)$$

Accordingly, NEE*_*i*_* is flagged as spike if:


(2) $$d_{i}<M\,d-\left(z\cdot M\,A\,D/0.6745\right)$$

or


(3) $$d_{i}=M\,d+\left(z\cdot M\,A\,D/0.6745\right)$$

where


(4) $$\text{MAD}=\text{median}\,\left(\left|d_{i}-M\,d\right|\right)$$

*Md* is the median of the differences; *z* is a threshold value, which equals to 5.5 in this study. Following the micrometeorology convention, negative NEE values indicate a net uptake of CO_2_ by the peatland while positive NEE values represent a net release to the atmosphere.

After applying the QA/QC criteria, ∼60% of the original measurements remained in the dataset. To obtain the NEE of a continuous time series, the REddyProcWeb online tool^2^ was used to fill gaps and to partition NEE into gross primary production (GPP) and ecosystem respiration (R_*eco*_) (Figure 5; Wutzler et al., 2018).

**FIGURE 2:**
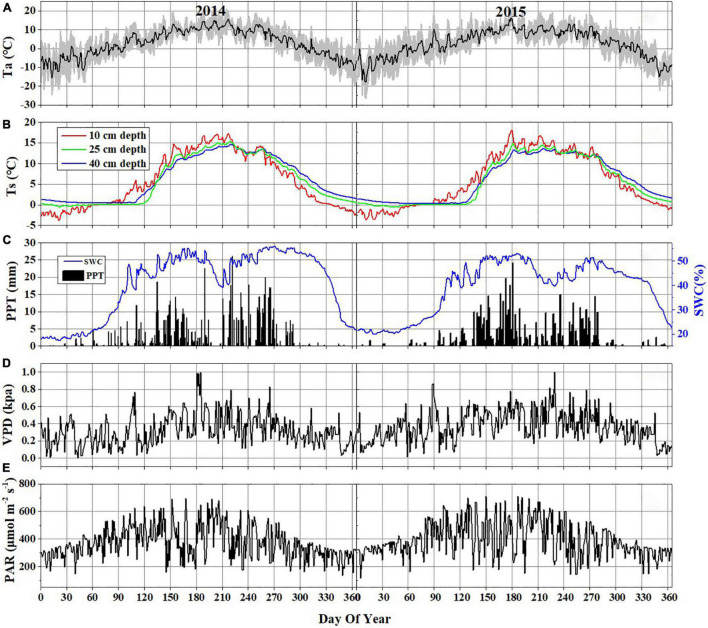
Daily variations of major environmental variables in 2014 and 2015: **(A)** air temperature (Ta) at 2 m above the ground (the black line represents daily means and the shaded area denotes standard deviation for each half-hour interval); **(B)** soil temperature (Ts) at 10 cm (blue line), 25 cm (green line), and 40 cm (red line) below the ground; **(C)** daily precipitation (PPT) and daily mean soil water content (SWC) at 10 cm depth below the ground; **(D)** daily mean VPD; **(E)** daily mean PAR.

**FIGURE 3:**
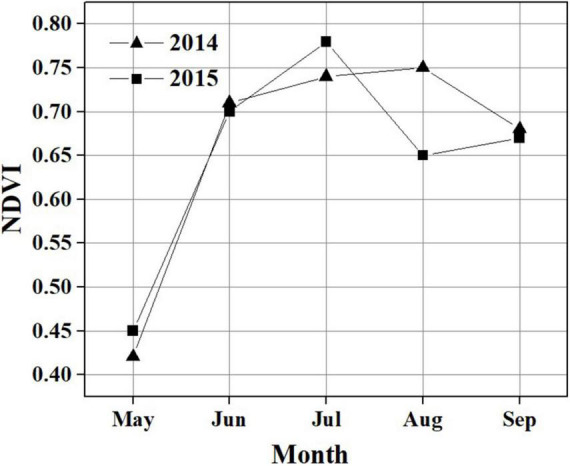
Monthly variation in NDVI during the growing season in 2014 and 2015.

**FIGURE 4:**
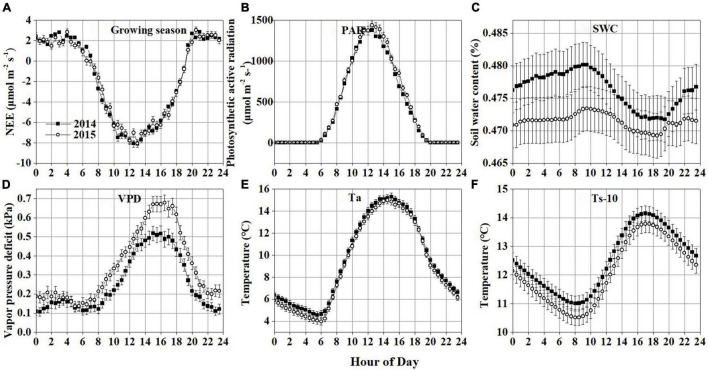
Average diurnal variations during the growing season in 2014 and 2015: **(A)** net ecosystem CO_2_ exchange (NEE) during the growing season; **(B)** photosynthetically active radiation (PAR); **(C)** soil water content (SWC); **(D)** vapor pressure deficit (VPD); **(E)** air temperature (Ta); **(F)** soil temperature at 10 cm depth below the ground (Ts-10). Error bars in all plots denote the standard errors. The time axes in the graph record Beijing time (UTC+8).

**FIGURE 5:**
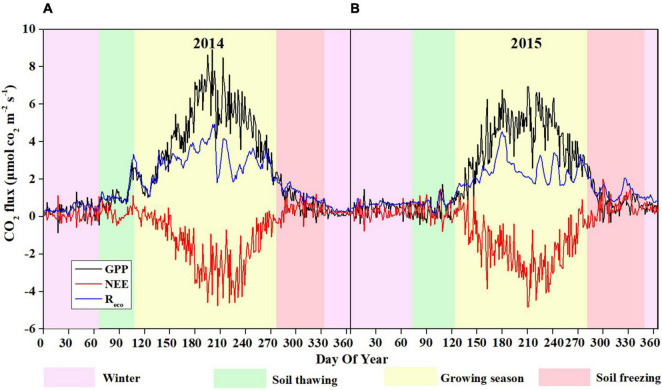
Seasonal patterns of daily NEE, GPP, and R_*eco*_ in 2014 **(A)** and 2015 **(B)**.

The Q_10_ value is used to describe the magnitude of the respiratory rate variation with a temperature change of 10°C. The Q_10_ based equation is as follows (Reichstein et al., 2002):


(5) $$Q_{10}={\text{e}}^{10\,b}$$

Where *b* is obtained from the model *R_eco_* = *ae^bT^* (Xu and Baldocchi, 2004). *T* is the daily average soil temperature (at a depth of 10 cm below the ground) during the nighttime; *R*_*eco*_ is the daily averaged ecosystem respiration.

The Normalized Difference Vegetation Index (NDVI) has a good correlation with vegetation leaf area index (LAI) and biomass (Deng et al., 2006; Zhang et al., 2010); therefore, the NDVI can be used as an indicator of green area in Landsat imagery. NDVI was obtained from Landsat surface reflectance product [Landsat 7 ETM+ (30 m) and Landsat 8 OLI (30 m)], downloaded from the United States Geological Survey (USGS) EarthExplorer^3^. The Landsat imagery has been preprocessed with atmospheric correction and topographic correction before the analysis (Hwang et al., 2011). The effect of clouds and cirrus on reflectance was excluded (Zhou et al., 2016).

### The Division of Seasons

In order to explore the seasonal variation of CO_2_ flux, the seasons were divided into four time periods according to different biophysical conditions, including (1) the growing season, which starts from the first day of seven consecutive days when the daily average Ta is higher than 5°C and ends on the first day of seven consecutive days when the daily average Ta is lower than 5°C (Lund et al., 2010), (2) soil freezing, from the last day of the growing season to a day when the daily average Ts of two consecutive days is lower than 0°C, (3) soil thawing, between the growing season and winter (Lund et al., 2010), and (4) winter, from the last day of the soil freezing period to the day when the Ts is above 0°C. Non-growing season comprises soil thawing, soil freezing, and winter periods (Table 1).

**TABLE 1:** Cumulative NEE, GPP, and R_*eco*_ and daily mean of NEE, GPP, and R_eco_ (g C m^–2^) in 2014 and 2015.

Period	Year	Total NEE	Total GPP	Total R_eco_	Daily mean NEE	Daily mean GPP	Daily mean R_eco_	Number of days
Growing season	2014	–279.96	783.55	503.59	–1.64	4.58	2.95	171
	2015	–260.43	649.66	389.23	–1.68	4.19	2.51	155
Soil freezing	2014	23.56	57.09	80.65	0.38	0.92	1.30	62
	2015	35.53	64.32	99.85	0.50	0.90	1.40	71
Winter	2014	22.16	23.46	45.62	0.24	0.25	0.48	94
	2015	21.82	42.16	63.98	0.25	0.48	0.73	87
Soil thawing	2014	7.63	33.88	41.51	0.20	0.89	1.09	38
	2015	17.73	20.70	38.43	0.34	0.40	0.88	52

### Wavelet Analysis

Continuous wavelet transform (CWT) with the Morlet wavelet was used to investigate the spectral characteristics of NEE and environmental variables. The Morlet wavelet is composed of a real part and an imaginary part, which makes it easy to analyze amplitude and phase, respectively (Grinsted et al., 2004). Wavelet spectral analysis is a more powerful tool for analyzing time series with non-stationary (including trace-gas flux data measured by eddy covariance), compared to the Fourier analysis (Vargas et al., 2010; Hatala et al., 2012). The wavelet coherence spectrum is expressed as the local correlation between two variables in frequency-time space, and its high coherence indicates a phase-locked behavior between two time series (Grinsted et al., 2004). For time periods with significant wavelet coherence, we used the phase angle to judge the time lag between the correlation oscillations of the two sequences (Hatala et al., 2012). To avoid spurious correlations between NEE and environmental variables, missing values were filled with median values before and after the gap. All wavelet analyses were performed in MATLAB (R2016a, MathWorks Inc., United States).

## Results

### Environmental Conditions

During the observation period, the Hongyuan peatland was characterized by strong variations in temperature (Ta and Ts), SWC, PPT, VPD, and photosynthetically active radiation (PAR) (Figure 2). Most of precipitation in this area occurred during the growing season when the sunshine was abundant and temperature was high (Figures 2B,C). Such climate conditions are conducive to plant growth and residue accumulation, which is beneficial for the development of peatlands. The daily mean Ta ranged from −15.6 to 15.8°C in 2014 and −18.1 to 16.4°C in 2015 (Figure 2A). The mean annual Ta was 2.8 and 2.7°C in 2014 and 2015, respectively. Ts at different depths showed similar seasonal patterns during the observation period, with its maximum values (at a depth of 10 cm) appearing around late June and early June in 2014 and 2015, respectively (Figure 2B). The daily mean PAR in the growing season was 420 μmol photons m^–2^ s^–1^ in 2014 and 443 μmol photons m^–2^ s^–1^ in 2015, which was higher than that in the non-growing season (338 μmol photons m^–2^ s^–1^ in 2014 and 358 μmol photons m^–2^ s^–1^ in 2015) (Figure 2E). The maximum monthly PAR of the growing season in both years occurred in July. Annual precipitation was 794.8 and 586.8 mm in 2014 and 2015, with most of the precipitation occurring during the growing season (Figure 2C). There also existed significant differences of monthly precipitation sums during the growing season in these 2 years. The maximum rainfall occurred in September of 2014 (191.4 mm) and in June of 2015 (174.8 mm). The variations in precipitation and temperature in these 2 years had obvious effects on SWC (Figures 2A,C). The seasonal pattern of SWC in 2014 was similar to that in 2015 (Figure 2C). However, a higher SWC value in 2014 was observed due to more precipitation than in 2015. The SWC remained below 20% before the rainy season began in late April in each year. Due to a sudden drop in rainfall in July 2014, the SWC decreased to ≤ 45%. The daily mean VPD exhibited similar seasonal patterns in both years, with high values (0.41 kPa in 2014 and 0.47 kPa in 2015) during the growing season and low values (0.24 kPa in 2014 and 0.29 kPa in 2015) during the non-growing season (Figure 2D). Overall, the VPD values in 2015 were higher than those in 2014. The maximum NDVI was 0.75 in August 2014 and 0.78 in July 2015. The NDVI from June to August in 2014 was significantly higher than that in 2015 (Figure 3). The mean NDVI was 0.658 and 0.65 during the growing season of 2014 and 2015, respectively.

### Diurnal Variations in Net Ecosystem CO_2_ Exchange

The NEE showed a clear diurnal pattern during the non-growing season in 2014 and 2015, with a negative value occurring between 10:00–16:00 (Supplementary Figure 1A). The CO_2_ flux in this period varied from −0.64 ± 0.19 (mean ± standard error) to 0.84 ± 0.13 μmol m^–2^ s^–1^ in 2014 and from −0.65 ± 0.21 to 1.28 ± 0.17 μmol m^–2^ s^–1^ in 2015. This may be attributed to that weak photosynthesis still existed in the early stage of soil freezing. However, due to the short duration and small absolute value of NEE, the Hongyuan peatland acted as a weak carbon source during this period. In the growing seasons, the NEE showed a significant diurnal variation pattern, with a negative value occurring between 7:00–19:00 (Figure 4A), during which the CO_2_ uptake rate started to increase at around 7:00, reached a peak value of −8.1 ± 0.34 μmol m^–2^ s^–1^ at 12:30, and then decreased to −0.75 ± 0.11 μmol m^–2^ s^–1^ at 19:00. After 19:00, the Hongyaun peatland started to release CO_2_ into the atmosphere, with a peak value of 2.8 ± 0.07 μmol m^–2^ s^–1^ occurring at 21:00. Clear diurnal variation patterns were also observed in PAR (Figure 4B), SWC (Figure 4C), VPD (Figure 4D), Ta (Figure 4E), and Ts-10 (Figure 4F) in these 2 years. But there are no strong diurnal patterns in Ts-25 (Supplementary Figure 1B) and Ts-40 (Supplementary Figure 1C). The peak NEE value appeared earlier than VPD, Ta and Ts, later than SWC, but coincides with PAR. Nonetheless, these diurnal variation patterns are calculated based on the average and standard error of the seasonal time scale, and thus cannot represent their real-time fluctuations. Therefore, wavelet analysis is used to reveal short-time scale changes in NEE and environmental variables.

### Seasonal Variations in Net Ecosystem CO_2_ Exchange

The seasonal variations in NEE showed similar patterns in 2014 and 2015 (Figure 5), with the maximum CO_2_ uptake (−4.92 and −4.96 g C m^–2^ d^–1^ in 2014 and 2015, respectively) in late July, and the minimum CO_2_ uptake (0.001 and 0.003 g C m^–2^ d^–1^ in 2014 and 2015, respectively) in early January, which are consistent with the variations in Ta and Ts-10 (Figures 2A,B). During the growing seasons, the daily NEE became slightly negative since late May, reached minimum value in late July, and then increased through August and September. However, there were more days (171 days) during the growing season in 2014 than those in 2015 (155 days) (Table 1). Thus, the total NEE during the growing season in 2014 (−279.96 g C m^–2^) was higher than that in 2015 (−260.43 g C m^–2^) (Table 1). No significant fluctuation in CO_2_ flux was observed during soil freezing, winter and soil thawing (Figure 5). At the beginning of rainy season in 2014 and 2015, both GPP and R_*eco*_ started to increase significantly. Daily R_*eco*_ displayed relatively smooth seasonal pattern with the range of 1.09–5.11 and 0.94–4.7 g C m^–2^ d^–1^ during the growing seasons in 2014 and 2015, respectively. GPP showed a similar seasonal variation pattern to NEE. The maximum daily GPP occurred in July, with the values of 7.2 g C m^–2^ d^–1^ in 2014 and 9.2 g C m^–2^ d^–1^ in 2015.

During the soil freezing period, the cumulative NEE was 23.56 g C m^–2^ in 2014 and 35.53 g C m^–2^ in 2015. In the winter, the NEE remained at a constantly low level, with mean values of 0.24 and 0.25 g C m^–2^ d^–1^ in 2014 and 2015, respectively. And the cumulative NEE was 22.16 g C m^–2^ in 2014 and 21.82 g C m^–2^ in 2015. In the soil thawing period, the daily mean NEE was 0.20 and 0.34 g C m^–2^ d^–1^ in 2014 and 2015, respectively. The cumulative NEE, R_*eco*_, and GPP over the 2 years were −226.61, 671.37, and 897.98 g C m^–2^ in 2014, and −185.35, 591.49, and 776.84 g C m^–2^ in 2015 (Supplementary Figure 2).

### Variations in Net Ecosystem CO_2_ Exchange and Environmental Factors in the Time-Frequency Domain

**FIGURE 6:**
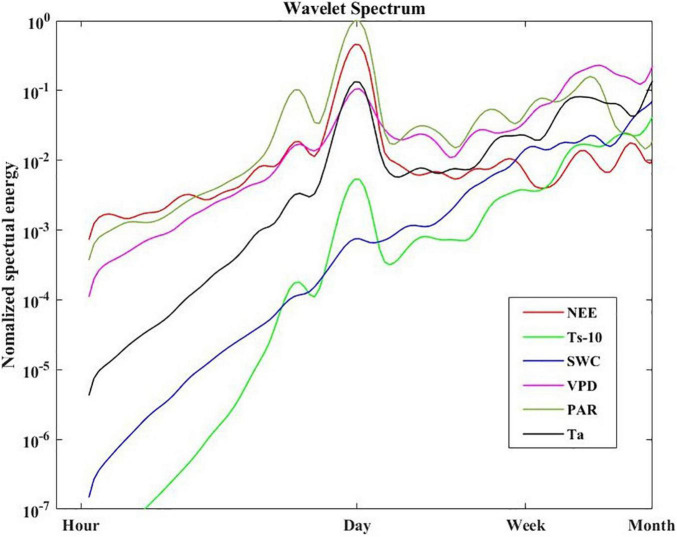
Normalized global wavelet energy spectra for NEE, Ts-10, SWC, VPD, PAR, Ta.

The global wavelet power spectral of NEE, Ts-10, VPD, PAR and Ta all showed strong peaks at 1-day period (Figure 6). But a weak spectral energy peak was observed for SWC. The PAR also displayed a clear peak at the 0.5-day (i.e., diurnal) period, but the spectral energy peak was much lower than that at the 1-day period. Meanwhile, the PAR had relatively higher spectral energy peaks than other variables on the daily scale.

**FIGURE 7:**
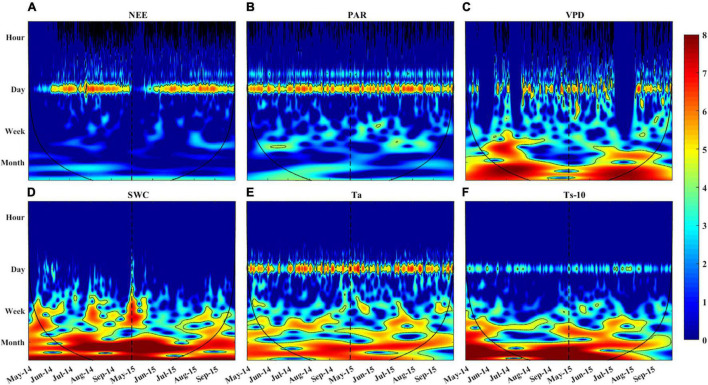
Wavelet power spectrum for **(A)** NEE, **(B)** PAR, **(C)** VPD, **(D)** SWC, **(E)** Ta, and **(F)** Ts-10 during the growing season in 2014 and 2015. The black line represents the cone of influence, beyond which edge effects are abundant and thus the results should be interpreted with caution. The color codes for power values are from dark blue (low values) to dark red (high values). The dotted line is used to distinguish the growing season of 2014 and 2015.

Continuous wavelet transformation has revealed the local characteristics of NEE and environmental variables in the time-frequency domain (Figure 7). The diurnal variation of NEE was inconsistent throughout the growing season. The NEE oscillated strongly at 1-day period from early June to late September, while it oscillated weakly in May (Figure 7A). The areas of the significant wavelet power for PAR, VPD, SWC, Ta, and Ts (Figures 7B–F) corresponded to their spectral peaks in Figure 6. We noted that the SWC, Ta, and Ts oscillated strongly at periods between weekly to monthly as indicated in the time-frequency domain.

**FIGURE 8:**
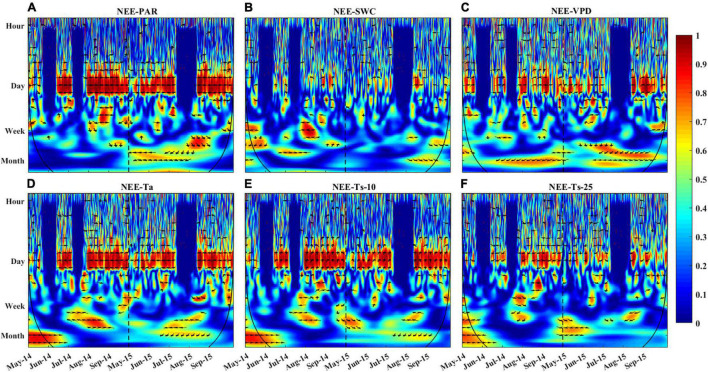
Wavelet coherence between NEE and **(A)** PAR, **(B)** SWC, **(C)** VPD, **(D)** Ta, **(E)** Ts-10, and **(F)** Ts-25. Red color indicates a high correlation between NEE and variables on the given time scale. The direction of arrows represents phase shift between NEE and associated variables (right: NEE and variables are in phase, which means the series are perfectly correlated; up: NEE leads variable by π/2; left: NEE and variables are out of phase; down: NEE lags variable by π/2). The black line represents the cone of influence, beyond which edge effects are abundant and results should be interpreted with caution. The dotted line is used to distinguish the growing season of 2014 and 2015.

The relationship between NEE and single environmental variable in the time-frequency domain could be described using the wavelet coherence (Figure 8). The patterns of correlation between NEE and diverse variables (PAR, VPD, Ta, and Ts) were similar. However, the phase shift between the NEE flux and these variables was different, especially on shorter timescales. For example, all environmental variables showed obvious correlations with NEE on daily time scale, but only the fluctuations of PAR and NEE were synchronized (Figure 8A). Besides, the VPD, Ta, SWC, and Ts (Figures 8B–F) were all correlated with NEE from daily to seasonal timescales. But strong coherencies occurred at the daily timescales, which were maintained for the duration of the growing season.

## Discussion

### Diurnal Variation in Net Ecosystem CO_2_ Exchange

The diurnal variation of NEE generally shows different patterns and magnitudes due to the diversity of vegetation types, climate conditions and underlying surface conditions (Vitale et al., 2007; Han et al., 2020; Zhu et al., 2020; D’Angelo et al., 2021). For example, a significant bimodal diurnal variation was observed in the coastal zone, where nearshore water served as an atmospheric CO_2_ sink throughout the day (Chien et al., 2018). Previous studies have found that the pattern and magnitude of NEE were strongly regulated by Rg, VPD, and water use efficiency, which are important drivers of plant photosynthesis (Chapin et al., 2002; Vitale et al., 2007). In this study, a clear unimodal diurnal variation pattern occurred in the Hongyuan peatland during the observation period. This diurnal variation pattern is related to the variations in total solar radiation and PAR induced by the solar cycle throughout the day (Stoy et al., 2005; Zhao et al., 2006; Ouyang et al., 2014). Specially, the change of the solar altitude angle will shorten or extend the length of daylight. When the solar altitude angle is high and the PAR is strong (such as June and July) (Figure 2E), the duration of positive value will be short, while the duration of negative value will be long. On the other hand, during the early and late periods of the growing season (such as May or September) when the solar altitude angle is relatively low and the PAR is weak, the duration of the positive values will be longer, and the duration of the negative values will be shorter (Ouyang et al., 2014).

Wavelet coherence analysis showed that NEE was more tightly correlated with PAR than other environmental factors at the daily time scale (Figure 8A). PAR has more oscillating power than other environmental variables at the daily scale (Figure 6), which may transmit more variation to ecosystem function (e.g., NEE). Besides, the daily course of NEE precedes that of VPD, Ta, Ts-10 (Figures 8C–E), but lags that of Ts-25 (Figure 8F). Thus, PAR was considered to be the primary driver for NEE during the growing season at the diurnal time scale. Previous studies have also demonstrated that carbon absorption is largely affected by PAR (Yang et al., 2011; Jia et al., 2014), whereas carbon release is mainly controlled by temperature (Sha et al., 2021).

In addition, the maximum CO_2_ uptake in the Hongyuan peatland was compared with that observed in other sites located at similar latitudes (Supplementary Table 1). We found that the maximum CO_2_ uptake of the Hongyuan peatland (−12.3 and −12.2 μmol m^–2^ s^–1^ in 2014 and 2015, respectively) was similar to that measured in an alpine wetland in Southwest China (−12.3 μmol m^–2^ s^–1^, Hao et al., 2011), but was higher than those in alpine steppe ecosystems (−3.44 μmol m^–2^ s^–1^, Zhu et al., 2015; −3.4 μmol m^–2^ s^–1^, Wang et al., 2018) and in alpine meadow ecosystems on the QTP (−3.74 μmol m^–2^ s^–1^, Zhao et al., 2010; −8.3 μmol m^–2^ s^–1^, Shi et al., 2006). This indicates that the alpine peatland has a higher potential of CO_2_ uptake than the alpine meadow and alpine steppe ecosystems at similar elevation and latitude, which may be linked to aboveground biomass. The average aboveground biomass for the Ruoergai alpine peatland was 340 g m^–2^ (Ma et al., 2017), which was higher than that in alpine meadow (≤151 g m^–2^, Shi et al., 2006) and alpine steppe (≤108 g m^–2^, Zhu et al., 2015) ecosystems on the QTP. Han et al. (2013) also found that aboveground biomass showed significant negative relationship with NEE over a reed wetland. This probably occurred because the direct and indirect effects of biomass on plant physiological metabolism process through the photosynthesis and respiration. Firstly, to some extent, aboveground biomass was associated with photosynthetic capacity of plant (Han et al., 2013), therefore NEE was regulated by the amount of plant biomass (Larmola et al., 2003). Secondly, biomass is a good proxy for accounting for variation in both autotrophic and heterotrophic capacity for respiration (Tong et al., 2017) and therefore the variation in aboveground biomass regulated the variability in ecosystem respiration (Wohlfahrt et al., 2008). Lastly, aboveground biomass was related to the leaf area index (Wickland et al., 2001), which could affect NEE by controlling ecosystem light absorption capacity (Lund et al., 2010).

### Seasonal and Interannual Variation in Net Ecosystem CO_2_ Exchange

Due to the limitations of climate conditions and observation instruments, less attention has been paid to the CO_2_ exchange during the non-growing season (Zhu et al., 2015; Shang et al., 2016; Xu et al., 2020). In this study, the annual NEE during the growing season in 2014 and 2015 was 1.2 times and 1.4 times of the annual total, respectively, indicating that the net carbon emission (53.35 g C m^–2^ in 2014 and 75.08 g C m^–2^ in 2015) during the non-growing seasons could not be ignored (Table 1). Considering that the total area of the undisturbed peatland in the Ruoergai region is 3,179 km^2^ (Chen et al., 2014), the estimated total CO_2_-C emission from the Ruoergai peatland during the non-growing season is about 0.17 Tg C, implying that the CO_2_ flux in the non-growing season could not be ignored. The Q_10_ value during the non-growing season was estimated to be 3.32 in both 2014 and 2015, which was higher than that during the growing season (2.58 and 2.22 in 2014 and 2015, respectively). This suggests that CO_2_ exchange during the non-growing season is more sensitive to warming than that in the growing season.

The length of the growing season had an important effect on CO_2_ sequestration (Churkina et al., 2005; Loisel et al., 2012). Some studies found that a longer growing season resulted in a greater CO_2_ uptake (Baldocchi et al., 2001; Euskirchen et al., 2006). However, others showed that a longer growing season lead to the net carbon loss of ecosystem (Hu et al., 2010; Piao et al., 2011; Wu et al., 2013). Our data revealed that the longer the growing season was, the greater the magnitude of CO_2_ uptake became. In the Hongyuan peatland, the CO_2_ uptake of the ecosystem would increase by 1.2 g C m^–2^ for each additional day in the growing season. Similar phenomenon was also observed by Wu et al. (2020) over an alpine meadow in the northeastern edge of the QTP, China. Various biophysical factors (such as Ts, Ta, phenology) can influence the length of the growing season, and the factors are different in different kinds of ecosystems (Lund et al., 2010; Garrity et al., 2011). In the Hongyuan peatland, we thought that the Ts was the dominant factor. The average diurnal Ts in 2014 was about 0.42°C higher than that in 2015, resulting in the growing season in 2014 to arrive 2 weeks earlier than in 2015. This may be primarily due to earlier thaw of soil driven by higher temperature, which accelerated the vegetation activity (Euskirchen et al., 2006).

The inter-annual variation of NEE in wetland ecosystems could be affected by many factors, which vary with climate conditions or vegetation styles (Lafleur et al., 2003; Hao et al., 2011; Cao et al., 2017). In the Hongyuan peatland, the annual PPT was likely to be the main factor controlling the inter-annual variation of NEE. The annual PPT in 2014 was 35% more than that in 2015, and the ecosystem in 2014 fixed 22% more carbon than in 2015. This may be caused by the wet conditions that could increase the photosynthesis of vegetation and thus promoted the carbon sequestration capacity in an ecosystem (Trudeau et al., 2014). Other studies have also proposed that the carbon cycle in wetland ecosystems was dominated by PPT (Griffis and Rouse, 2001; Aslan-Sungur et al., 2016; Drollinger et al., 2019). A significant negative correlation of NEE with PPT in an arid desert wetland ecosystem has been established by Gou et al. (2017). And higher CO_2_ uptake has been measured in a sedge fen during wet years, which was attributed to the higher photosynthetic capacity of the vegetation in wet years (Aurela et al., 2007). Sonnentag et al. (2010) suggested that the inter-annual variability of CO_2_ fluxes in a minerotrophic fen was mainly controlled by soil water content and water table level.

### Effect of Temperature on Net Ecosystem CO_2_ Exchange

**FIGURE 9:**
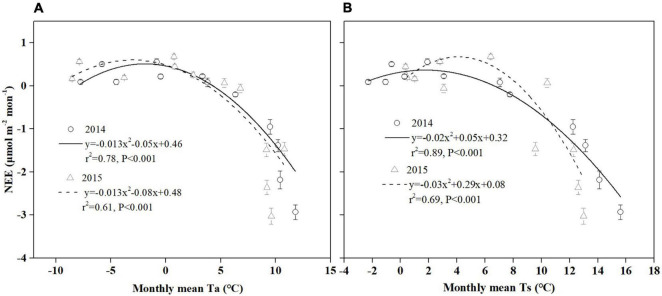
The monthly relationship between NEE and temperature [**(A)** Ta, **(B)** Ts] during 2014 and 2015 in the Hongyuan peatland ecosystem. Error bars in all plots denote the standard errors.

**FIGURE 10:**
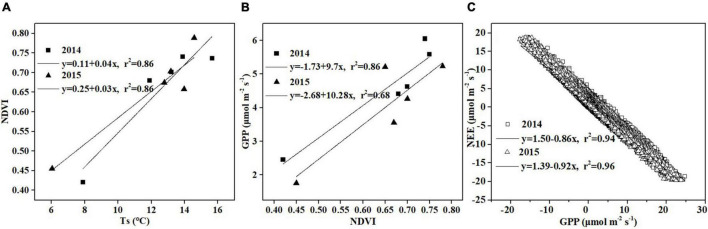
Relationship between **(A)** Ts and NDVI, **(B)** NDVI and GPP, and **(C)** GPP and NEE.

Temperature has a strong effect on photosynthesis and carbon sequestration of vegetation in alpine peatland ecosystem (Hao et al., 2011; Kang et al., 2018). Temperature has been thought to be one of the key environmental factors controlling the photosynthesis rate as the temperature has an important impact on plant dormancy, leaf phenology and late growth (Ueyama et al., 2013). In addition, temperature can influence soil microbial activity, enzyme activity and organic matter decomposition (Zhu et al., 2020). Our results showed that a significant correlation was observed between temperature (Ts and Ta) and NEE in Hongyuan alpine peatland, and higher temperature increased CO_2_ uptake (Figure 9). The Ta and Ts could explain 70 and 79% of the variations in NEE, respectively, indicating that Ts has a stronger effect on CO_2_ exchange in the Hongyaun peatland. Although previous studies of the CO_2_ exchange in alpine wetlands have mentioned the importance of temperature (Cao et al., 2017; Zhu et al., 2020), such a clear correlation between NEE and Ts has not been described. The high dependence of NEE on Ts could be related to two factors. First, the belowground biomass accounts for a larger proportion relative to the aboveground biomass. In Ruoergai peatland, the belowground biomass (3,262.93 g m^–2^) was about 10 times the aboveground biomass (341.01 g m^–2^) (Ma et al., 2017). The fact that a large proportion of belowground biomass may lead to a high correlation between Ts and NEE (Ma et al., 2017). Second, the increase in Ts could cause the elevation of NDVI (Figure 10A), which in turn leads to high GPP (Figure 10B). Similar pattern was also observed over the northern high latitudes (Myneni et al., 1997) and the QTP (Shen et al., 2011). Our observation showed that NEE has a negative relationship with GPP, indicating that the ecosystem with higher GPP possibly has greater net CO_2_ uptake (Figure 10C). This could be explained by the fact that high Ts can promote vegetation growth, thereby improving the photosynthesis and carbon sequestration capacity of vegetation.

### Effects of Rain Pulses on Net Ecosystem CO_2_ Exchange

**FIGURE 11:**
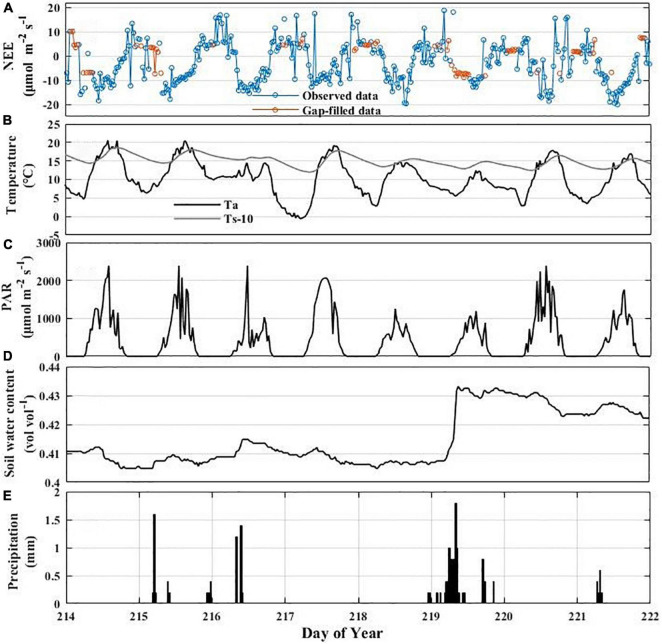
Variation in NEE and environmental variables associated with rainfall events: synoptic variations of half-hourly NEE **(A)**, air temperature and soil temperature at 10 cm depth **(B)**, PAR **(C)**, soil water content (SWC) **(D)**, and precipitation (PPT) **(E)**.

The impact of rain pulses on NEE has also been well documented in many ecosystems (Aires et al., 2008; Hao et al., 2011). For example, Chou et al. (2008) suggested that the rain pulse had a crucial effect on regulating the CO_2_ balance in grassland ecosystem. In this study, we found that rainfall events triggered the pulse dynamics of NEE in the Hongyuan peatland ecosystem (Figure 11). NEE started to increase significantly and even reached a positive value once the heavy rain occurred on DOY 219 (10 mm) (Figures 11A,E). Our result is consistent with experimental data in an alpine wetland on the QTP of Zhu et al. (2020) that found the increase of precipitation could accelerate the carbon loss. This may be caused by that rainwater penetration can quickly make the physical environment favorable to microbes and that the soil water produced by the rain pulse may displace the CO_2_ stored in the soil pores. Another reason may be the decomposition, mineralization and release of inorganic carbon when the soil dries out due to lack of rain in summer and then is rewetted by precipitation (Jarvis et al., 2007).

The CO_2_ uptake reached its maximum value 1–2 days after the rainfall events on DOY 219 and DOY 215–216 (Figures 11A,E). This may be linked to the environmental conditions that are optimum for carbon assimilation after rainfall events (Figures 11B–D). This finding agrees with the observations in other ecosystems. Jia et al. (2014) observed that the NEE peak lagged behind the rain pulse by 1–2 days in a semiarid shrubland. Similarly, it took 4–5 days to reach the maximum value after the rainfall events in a saline desert ecosystem (Liu et al., 2012). Furthermore, Aires et al. (2008) observed that heavy rainfall events could lead to the growth of C_4_ grasses and the subsequent CO_2_ sequestration in the Mediterranean grassland. Jarvis et al. (2007) has also confirmed that the enhancement of the ecosystem respiration and the hysteresis of the maximum NEE during rainfall events may be caused by the rapid response of soil organic matter and microorganisms. It is noteworthy that not all rain pulses have the same impacts on NEE (Figure 11A). For example, NEE was relatively insensitive to a smaller rainfall event on DOY 221 (1.4 mm). This may be ascribed to other biophysical factors that confound the NEE response to sudden increases in water availability (Chen et al., 2009). Compared with the rainfall events on DOY 219, the rainfall events on DOY 221 have less effect on Ta, Ts, SWC, and PAR, which could explain the result that the DOY 221 rainfall events did not cause a large fluctuation in NEE.

## Conclusion

This paper investigated the CO_2_ fluxes observed during a 2-year period using the open-path eddy covariance method over an alpine peatland on the eastern Qinghai-Tibetan Plateau. Our results show that the Hongyuan alpine peatland was a CO_2_ sink with an annual NEE of −226.61 and −185.35 g C m^–2^ in 2014 and 2015, respectively. The non-growing season NEE was 53.35 and 75.08 g C m^–2^ in 2014 and 2015, suggesting that non-growing seasons carbon emissions should not be neglected. Diurnal variation in NEE was observed during the growing season, with peak flux being recorded at 12:30 daily. In addition, we analyzed multi-scale dynamics and environmental controls on NEE in Hongyuan peatland. The spectral analysis showed how multiple environmental factors interact on different timescales and how their relative importance shifts during the growing season. PAR was the primary controlling factor of NEE on daily timescales. Seasonal variation in NEE was mainly driven by Ts. PPT was likely to be the main factor regulating the inter-annual variation in NEE. Our results provide important data for quantifying and modeling regional and global CO_2_ budget from alpine peatlands.

## Data Availability Statement

The original contributions presented in the study are included in the article/Supplementary Material, further inquiries can be directed to the corresponding author/s.

## Author Contributions

HY performed conceptualization and writing – original draft. HP and BH performed conceptualization and writing – review and editing. YH and YZ performed conceptualization and resources. QG performed methodology. HD performed data curation. CC performed software. JC performed writing – review and editing. All authors contributed to the article and approved the submitted version.

## Conflict of Interest

The authors declare that the research was conducted in the absence of any commercial or financial relationships that could be construed as a potential conflict of interest.

## Publisher’s Note

All claims expressed in this article are solely those of the authors and do not necessarily represent those of their affiliated organizations, or those of the publisher, the editors and the reviewers. Any product that may be evaluated in this article, or claim that may be made by its manufacturer, is not guaranteed or endorsed by the publisher.

## Footnotes

1
http://data.cma.cn/

2
https://www.bgc-jena.mpg.de/bgi/index.php/Services/REddyProcWeb

3
http://earthexplorer.usgs.gov/
