# Supplementary material for: Environmental Controls on Multi-Scale Dynamics of Net Carbon Dioxide Exchange From an Alpine Peatland on the Eastern Qinghai-Tibet Plateau
Source: Front Plant Sci. 2022 Jan 5;12:791343. doi: 10.3389/fpls.2021.791343 (PMC8767066; doi:10.3389/fpls.2021.791343)
Supplement: Supplementary file 1 [file Table_1.pdf]

Table S1: Comparison of the maximum CO_2 uptake from our study and data observed in other sites located at similar latitudes.

Study location	Latitude	Period	Altitude (m)	Ecosystem type	The maximum CO_2 uptake ($\mu mol\ m^{-2}\ s^{-1}$)	Reference
Hongyuan, China	32°46′ N	2014-2015	3510	Alpine peatland	-12.3	This study
Ruoergai, China	33°56′ N	2008-2009	3430	Alpine wetland(bog)	-12.3	Hao et al.(2011)
Nam Co, China	30°46′ N	2008-2009	4730	Alpine steppe	-3.44	Zhu et al. (2015)
Bange, China	31°25′ N	2014-2015	4700	Alpine steppe	-3.4	Wang et al. (2016)
Haibei, China	37°35′ N	2003-2006	3520	Alpien wetland meadow	-3.74	zhao et al.(2010)
Dzmxung, China	30°25′ N	2003-2005	4333	Alpine steppe-kobresia meadow	-8.3	Shi et al.(2006)
