# Supplementary material for: Environmental Controls on Multi-Scale Dynamics of Net Carbon Dioxide Exchange From an Alpine Peatland on the Eastern Qinghai-Tibet Plateau
Source: Front Plant Sci. 2022 Jan 5;12:791343. doi: 10.3389/fpls.2021.791343 (PMC8767066; doi:10.3389/fpls.2021.791343)
Supplement: Supplementary file 2 [file Image_1.pdf]

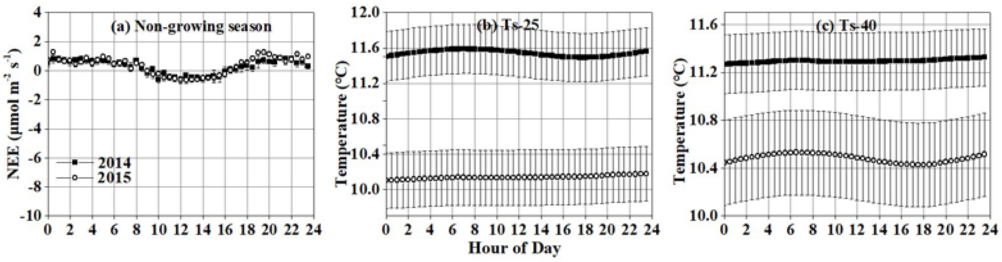

Figure S1 Average diurnal variations in 2014 and 2015: (a) net ecosystem CO_2 exchange (NEE) during the non-growing season; (b) soil temperature at 25 cm depth below the ground during the growing season; (c) soil temperature at 40 cm depth below the ground (Ts-40) during the growing season.
