# Supplementary figures and images for: Environmental Controls on Multi-Scale Dynamics of Net Carbon Dioxide Exchange From an Alpine Peatland on the Eastern Qinghai-Tibet Plateau
Source: Front Plant Sci. 2022 Jan 5;12:791343. doi: 10.3389/fpls.2021.791343 (PMC8767066; doi:10.3389/fpls.2021.791343)

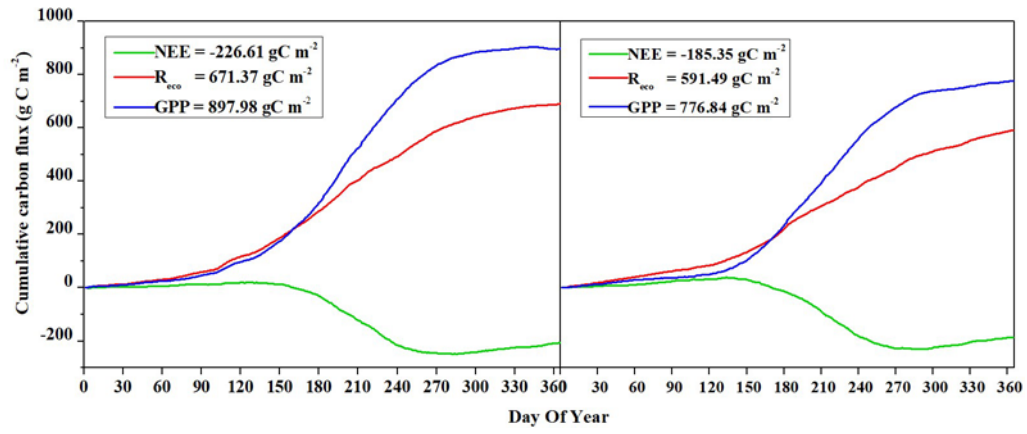

**Figure S2** cumulative NEE, R<sub>eco</sub>, and GPP in 2014 and 2015.

Supplement: Supplementary file 3 [file Image_2.pdf]
